# Effects of angle of incidence of stimulus light on photopic electroretinograms of zebrafish larvae

**DOI:** 10.1038/s41598-024-65017-0

**Published:** 2024-06-26

**Authors:** Hisashi Matsubara, Shinichiro Chujo, Yoko Mase, Yukiko Muramoto, Kumiko Kato, Mineo Kondo

**Affiliations:** Department of Ophthalmology, Mie University Graduate School of Medicine, 2-174 Edobashi, Tsu, Mie 514-8507 Japan

**Keywords:** Zebrafish, Electroretinography, ERG, Light stimulation, Fiber optical cable, Full-field stimulation, Electrophysiology, Neurophysiology, Biological techniques

## Abstract

In electroretinographic (ERG) recordings of zebrafish, the light stimulus is usually delivered by a fiber optic cable. The purpose of this study was to determine whether the angle of incidence of the stimulus light from the fiber optic cable will affect the amplitudes and implicit times of the ERGs of zebrafish larvae. The larvae were positioned on their side with the right eye pointed upward. The light stimuli were delivered by a fiber optic cable from three directions of the larvae: frontal 0° (F0°), dorsal 30°(D30°), and ventral 30°(V30°). Photopic ERGs were recorded from 16 larvae at age 5–6 days post-fertilization. Our results showed that the mean amplitude of the b-wave elicited at D30° and V30° stimulation was significantly smaller than that elicited at F0° stimulation (*P* = 0.014 and *P* = 0.019, respectively). In addition, the mean amplitude of the d-wave elicited at D30° and V30° stimulation was significantly smaller than that elicited at F0° stimulation (*P* < 0.0001 and *P* = 0.015, respectively). However, the difference between the b-wave amplitudes elicited at D30° and V30° stimuli were not significant (*P* = 0.98), and the d-wave amplitudes were also not significantly different (*P* = 0.20). The average b-wave amplitudes elicited at D30° stimulation was 84.6 ± 15.7% and V30° stimulation was 84.8 ± 17.4% relative to that of F0° stimulation. The average d-wave amplitudes elicited by D30° stimulation was 85.5 ± 15.2% and by V30° stimulation was 79.0 ± 11.0% relative to that of F0° stimulation. The differences in the implicit times of the b- and d-wave elicited by the different directions of stimulation were not significant *(P* = 0.52 and* P* = 0.14, respectively). We conclude that the amplitude of the photopic ERGs is affected by the angle of the incident light. Thus, it would be better to use ganzfeld stimuli to elicit maximum b- and d-wave amplitudes of the photopic ERGs of zebrafish larvae.

## Introduction

The zebrafish is an important animal model for various genetic and pharmacological studies on development because of their small size, manageable breeding schedule, transparency, availability, and cost^1^. Several methods have been used to assess the visual function of zebrafish, and electroretinography (ERG) has been generally used to evaluate the physiology and function of the retina of zebrafish. The ERGs evoked by light stimuli represent the changes in the electrical potential of the different neurons and their networks in the retina^2^. Earlier studies have shown that the implicit times and amplitudes of the ERGs were affected by the intensity of the light stimuli, the area of the retina stimulated, and the angle of the incident light stimuli on the photoreceptor cells^3–6^. Therefore, to evaluate the function of the entire retina, it is important to present the light uniformly across the retina to elicit responses from the entire retina. One method of accomplishing this is to use the ganzfeld stimulation method which is designed to stimulate the entire retina uniformly and is widely used for testing humans and animals. For ganzfeld stimulation, the eye is placed in a ganzfeld bowl, and the light stimulus is reflected from the inner surface of the bowl and stimulates the entire retina uniformly. In ERG recordings from zebrafish, the ganzfeld method or a light diffuser has been used to elicit reliable ERGs^7–9^. We have reported on the ERGs elicited by ganzfeld stimulation using light-emitting diodes as a light source^10^. However, in many ERG studies on zebrafish, the ganzfeld light stimulation method was not used. In such studies, many researchers have used a fiber optic cable placed in front of the eye to stimulate the eye. The fiber optic cable can be considered a unidirectional light source^11–15^. However, because zebrafish have small eyes and the ERG recording methods differ from that in mammals including the use of glass microelectrodes, the intensity of the light on the retina and the illuminated retinal area can easily be altered by the position of the zebrafish relative to the fiber optic bundle. As best we know, there have not been studies determining whether variations in the angle of incidence of the stimulus light affects the amplitudes and implicit times of the ERGs of zebrafish.

Thus, the purpose of this study was to determine the effects of the incidence of the light stimulus on the amplitudes and implicit times of the b- and d-waves of the photopic ERGs of zebrafish larvae. In addition, to determine a stimulation method to obtain repeatable recordings.

## Results

**Figure 1:**
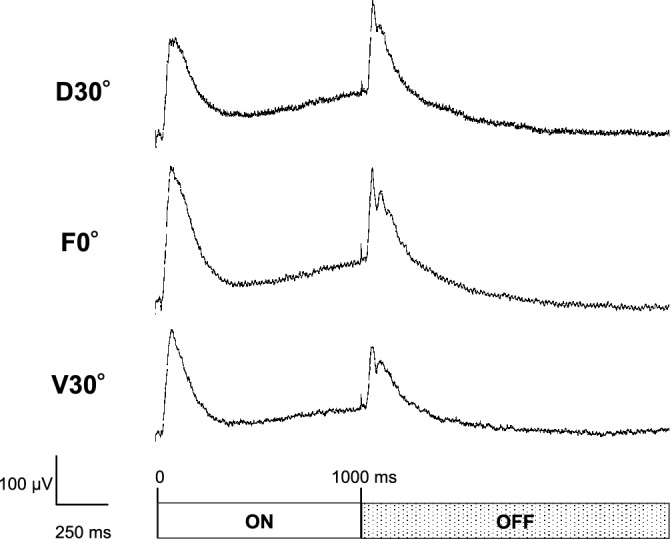
Representative electroretinograms (ERGs) elicited by a stimulus duration of 1000 ms from three different directions. Scale bars are shown on the images. D30°, dorsal 30°; F0°, frontal 0°; V30°, ventral 30°.

**Figure 2:**
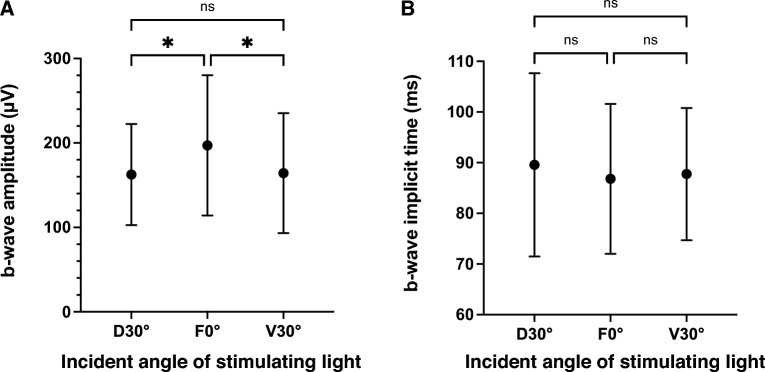
Means ± standard deviations of the amplitudes (**A**) and implicit times (**B**) of the b-waves elicited by the three different stimulus directions. Error bars represent the standard deviations. *P < 0.05; ns, not significant; one-way ANOVA with post-hoc Holm–Šídák's multiple comparison test. Exact *P values* are presented in the text. D30°, dorsal 30°; F0°, frontal 0°; V30°, ventral 30°.

**Figure 3:**
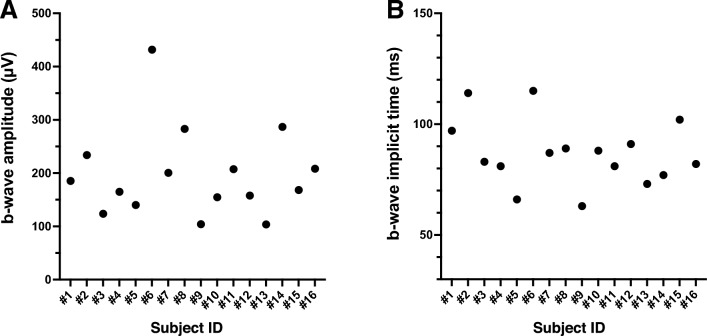
Plots of the b-wave amplitudes (**A**) and implicit times (**B**) of ERGs for F0° of all 16 larvae.

ERGs were recorded from 18 eyes of 18 larvae, and the ERGs consisted of the a-, b- and d-waves in all 18 eyes. Two eyes were excluded because the b-wave amplitudes were < 100 μV. Therefore, the data of 16 eyes of 16 larvae were statistically analyzed. Representative ERGs elicited by the three different stimulation angles are shown in Fig. 1. The mean amplitudes of the b-wave elicited by the different directions were 155.4 ± 65.1 μV from the dorsal 30° (D30°), 188.1 ± 88.6 μV from the frontal 0° (F0°), and 157.8 ± 73.7 μV from the ventral 30° (V30°). The b-wave amplitude at 0° was significantly larger than those at D30° and V30° (*P* = 0.014 and *P* = 0.019; Fig. 2A), but the differences between the amplitudes elicited by stimulation from D30° and V30° were not significant (*P* = 0.98; Fig. 2A). The relative b-wave amplitudes elicited by the D30° and V30° stimuli were 84.6 ± 15.7% (D30°) and 84.8 ± 17.4% (V30°) of the amplitude elicited by the F0° stimuli. The mean implicit time of the b-wave was 87.8 ± 18.9 ms at D30°, 85.1 ± 15.9 ms at F0°, and 87.1 ± 12.9 ms at V30°. None of the differences in the implicit times between the stimulation directions was significant (*P* = 0.52; one-way ANOVA; Fig. 2B). The coefficients of variation (CV) of the b-wave amplitude at F0° for the 16 larvae was 42.1% which was classified as high. The CV of the implicit times was 17.0% which was classified as moderate. Plots of the amplitudes and implicit times of the 16 larvae are shown in Fig. 3.

**Figure 4:**
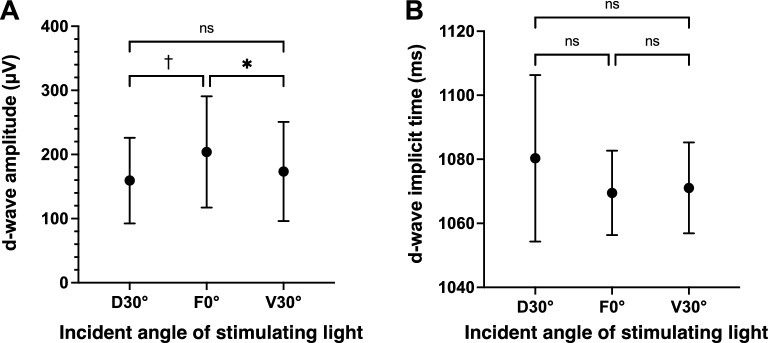
Means ± standard deviations of the amplitudes (**A**) and implicit times (**B**) of the d-waves for the three different stimulus directions. Error bars represent the standard deviations. *P < 0.05; ^†^*P* < 0.0001; *ns* not significant; one-way ANOVA with post-hoc Holm–Šídák's multiple comparison test. Exact *P* values are presented in the text. D30°, dorsal 30°; F0°, frontal 0°; V30°, ventral 30°.

**Figure 5:**
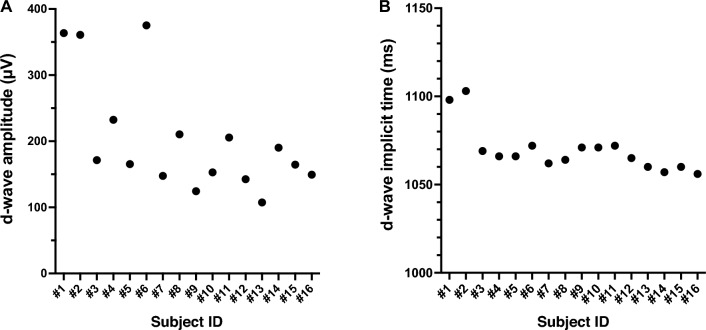
Plots of the d-wave amplitudes (**A**) and implicit times (**B**) of ERGs for F0° of all 16 larvae.

The mean amplitudes of the d-wave was 159.4 ± 66.8 μV at D30°, 204.0 ± 86.7 μV at F0°, and 173.4 ± 77.3 μV at V30°. The d-wave amplitudes at F0° were significantly larger than those at D30° and V30° (*P* < 0.0001 and *P* = 0.015; Fig. 4A), however the differences between the amplitudes elicited by stimulation from D30° and V30° were not significant (*P* = 0.20; Fig. 4A). The d-wave amplitudes elicited by the D30° and V30° stimuli were 85.5 ± 15.2% (D30°) and 79.0 ± 11.0% (V30°) of the amplitude elicited by the F0° stimuli. The mean implicit times of the d-wave was 1080 ± 26.0 ms at D30°, 1070 ± 13.2 ms at F0°, and 1071 ± 14.2 ms at V30°. None of the differences in the implicit times between the stimulation directions was significant (*P* = 0.14; one-way ANOVA; Fig. 4B). The CV of the d-wave amplitude at F0° among the 16 larvae was 42.5% which was classified as high. The CV of implicit time was 1.2% which was classified as low. Plots of the amplitudes and implicit times of the 16 larvae are shown in Fig. 5.

## Discussion

This study was designed to determine whether a difference in the angle of incidence of the stimulus light would affect the amplitudes and implicit times of the b- and d-waves of the larvae of zebrafish ERGs. The results showed that the amplitudes of the b- and d-wave were significantly smaller for the D30° and V30° stimuli compared to that of the F0° stimuli. However, the difference in the amplitudes at V30° and D30° was not significant. The amplitudes of the b- and d-waves of V30° and D30° were about 80% of the amplitude of the waves of F0°. The implicit time was not significantly different for the different angles of incidence of the stimulus light.

**Figure 6:**
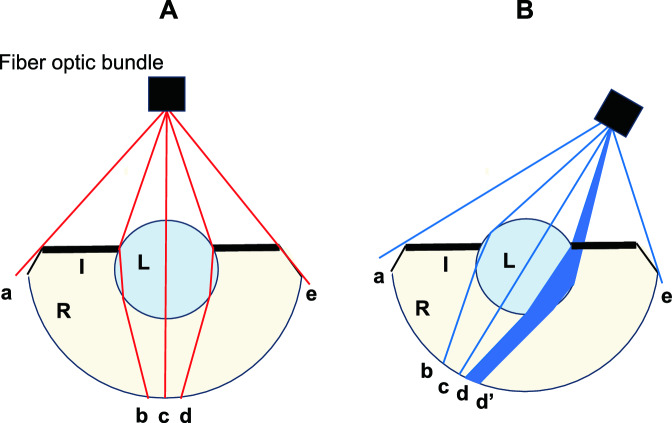
Schematic diagram of the light paths for different light incidences. This figure shows the differences in the retinal stimulated area due to differences in the stimulation direction. Reflected light by the cornea and lens is omitted. (**A**) frontal 0°, (**B**) lateral 30°. The retinal areas in a,b and d,e are not directly stimulated because the stimulating light does not reach those areas (**A**,**B**). The iris and the protruding retina block a part of the light from the 30° lateral directions. The blue-colored areas are the intercepted light fluxes. Then, the retinal area in d,dʹ is not directly stimulated (**B**). The area of the retina stimulated from frontal 0° is area in b–d is larger than that stimulated from lateral 30° area in b–d (**B**). I: iris, R: retina, L: lens.

Because the ERG is the sum of responses from the entire retina, the b-wave amplitude is greatly affected by the size of the retina receiving the light stimulation. Zebrafish larvae have a spherical and high refractive power crystalline lens that protrude from the pupil with no restriction from the iris. There is no gap between the lens and the retina, and the retina contacts the posterior surface of the lens^16^. In addition, the retina protrudes anteriorly from the equatorial part of the lens^16–18^. The light irradiating the entire eye is partially blocked by the pigmented iris and protruding retina. The stimulating light from the 0° direction passes through the pupillary region and enters the eye but does not directly stimulate the peripheral retina of the eye (Fig. 6A). On the other hand, the light from the 30° lateral directions enters the eye through the pupil, but the iris and the protruding retina block more of the light than the light from the 0° direction (Fig. 6B). Therefore, the amount of light reaching the retina will be lower when the incidence of the light is off axis, and the area of the retina directly stimulated by the light from 30° lateral is smaller than the area of the retina directly stimulated by the stimulating light from 0°. On the other hand, the light reflected from the cornea and lens may reach areas of the retina that were not stimulated by direct light from a light source. However, they are weaker than the direct stimulating light, and because the shape of the zebrafish lens is spherical, the difference in the amount of reflected light from the lens caused by the difference in angle of incidence is most likely lower.

The Stiles-Crawford effect is a phenomenon in which the retinal sensitivity is reduced by the angle of incidence of light on cone photoreceptor cells^19^. ERGs are affected by the Stiles-Crawford effect^20^. In the zebrafish retina, the cone photoreceptor cells are evenly distributed throughout the retina^21^, and there is little difference in refractivity depending on the direction of incidence because of the spherical lens shape. Therefore, there is little difference in the angle to the cone photoreceptor cells that the stimulus light stimulates, and the ERGs are not expected to be affected by the Stiles-Crawford Effect. The difference in stimulated area due to the large protruding area of the retina relative to the small size of the larval zebrafish eye may have the greatest effect on the amplitudes of the ERGs.

The implicit times of the ERGs are increased by a decrease in the intensity of stimulus light per unit area^22^. In an earlier study, some eyes with inherited retinal degenerations had prolongations of the implicit times before the changes in the amplitude^23^. In this study, the implicit times did not differ significantly for the off-axis stimulation but there were significant differences in the amplitudes. This suggested that the significant difference in the b- and d-wave amplitudes between the 0° and 30° lateral stimulus directions was due to the differences in the size of the stimulated areas.

There were differences in the amplitudes between the different larvae, and the CV for the amplitudes was about 40%. In an earlier study, we found that the CV of the b-wave amplitudes was 46.7% even with full-field stimulation^10^. These results indicated that the CV of the b-wave amplitude of the ERGs of larval zebrafish is high regardless of the stimulated area. On the other hand, the CV of the b-wave implicit time was moderate, and that of the d-wave was low. The exact reason for the high CV values in amplitudes and moderate or low CV values in the implicit times were not determined. The origins of the b- and d-waves are different with the b-wave originating from the activation of the ON pathway and the d-wave in the OFF pathway^24^. The ERG amplitudes and implicit times have been reported to change with maturity of the larvae^25^. Because the age range in this study was 5–6 days post-fertilization (dpf), there may have been differences in the maturity of the ON and OFF pathways among the individual larva. The ERG amplitude is affected by the immature development and minor mutations in wild-type zebrafish larvae^26^, which may be related to the individual differences in the b- and d-wave amplitudes. The differences in the cell number and function of the retina between the ON and OFF pathways may be related to the differences in the implicit times^27,28^.

This study has five limitations. The first limitation was that the larvae were selected only by excluding those with obvious behavioral defects and not by other methods such as the optokinetic reflex or optomotor responses. Therefore, larvae with congenital retinal abnormalities that could not be detected by superficial observations might have been included. The second limitation is that the age of larvae ranged between 5–6 dpf and was not fixed. This difference in age may have resulted in differences in the maturity. The third limitation is that the ERGs were only recorded at 30° lateral, not at angles smaller than 30°. Therefore, it is unclear how many degrees of displacement angle are acceptable for the ERG recordings of zebrafish larvae. The fourth limitation is that the distance from the eye to the optical fiber tip was fixed at 15 mm. Our recording system used glass microelectrodes. Therefore, the distance between the optical fiber and the eye was set at 15 mm to prevent interference between the glass microelectrodes and the fiber optic cable. The area of the retina to be stimulated could expand when the light source is brought closer but it was not tested whether this is possible due to its location relative to the electrode. We used a fiber optic cable with an outer diameter of 8 mm, but it may be possible to bring the light source closer by using a thinner fiber. As a next step, we need to plan studies to record ERGs by bringing the optical fiber tip closer to the eye. The fifth limitation is that only larvae were examined and not adults. In adult fish, the vitreous cavity between the posterior lens and the retina is formed, and the peripheral anterior projection of the retina decreases as it matures^18^. This morphological change may result in a smaller reduction in the area irradiated due to the lateral stimulation of the eye.

## Conclusions

The results indicate that the angle of incidence of the stimulating light affects the b-and d-wave amplitude of the ERGs of 5-6 days old zebrafish larvae. Therefore, the effect of the differences in the angle of incidence of the stimulating light cannot be eliminated in zebrafish ERG recordings using a light source not a full-field stimulation. It is best to use a full-field stimulator to eliminate uncertain factors such as variations in the angle of incidence of the stimulating light due to the placement of the subjects even in ERG recordings of zebrafish.

## Materials and methods

### Zebrafish lines and maintenance

The embryos of zebrafish, *Danio rerio*, of the RIKEN wild-type strain (RIKEN WT) were obtained from the National Bio Resource Project of the RIKEN Brain Science Institute (RIKEN, Saitama, Japan). The embryos and larvae were maintained under a 14-h light of approximately 500 lx on a 14:10 h light:dark cycle. The temperature of the aquarium E3 medium (5 mM NaCl, 0.17 mM KCl, 0.33 mM CaCl_2_, and 0.33 mM MgSO_4_) was 28 °C. After the completion of the experiments, the larvae were euthanized with a lethal dose of 3-aminobenzoic acid methyl ester (MESAB; Sigma, St. Louis, MO, USA).

### ERG recording procedures

**Figure 7:**
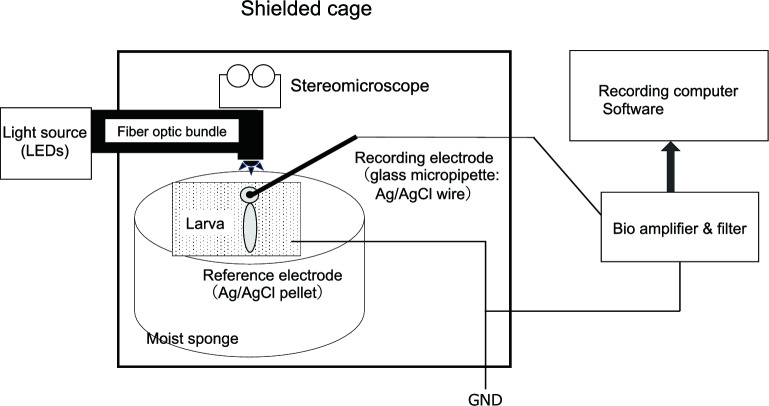
Schematic of the zebrafish ERG recording set-up. Body positioning and electrode setup of larvae were performed under a stereomicroscope. Light derived from the light-emitting diodes by optical fiber cable irradiated the front of the eye.

The setup and procedures for the ERG recordings were the same as reported in detail^10^. A glass micropipette with an opening of about 20 μm at the tip was filled with E3 medium, and a chlorided silver wire was inserted into the micropipette (Fig. 7). Then, the micropipette was fixed to a microelectrode holder (E45SW-F10PH, Warner Instruments, Hamden, CT, USA). This glass microelectrode (active electrode) was positioned on the center of the cornea while observing the procedure with a stereomicroscope. The reference electrode was a chlorided silver pellet placed under a moist paper towel resting on a sponge in a 35 mm Petri dish containing E3 medium. The chlorided silver wire in the microelectrode and the reference electrode were connected to a bioamplifier (AVB-10, Nihon Kohden, Tokyo, Japan). The electrical signals from the larva were differentially amplified 2000 times with bandpass cut-off frequencies of 0.8 and 300 Hz for all of the recordings. The amplified signals were fed to a PowerLab 2/25 instrument (AD Instruments Pty. Ltd., South Wales, Australia) using the Scope version 4.1 software (AD Instruments Pty. Ltd.), and the data acquisition, storage, and analyses were performed with a personal computer (iMac^Ⓡ^; Apple Computer, Inc., Cupertino, CA, USA).

### Light stimulation

**Figure 8:**
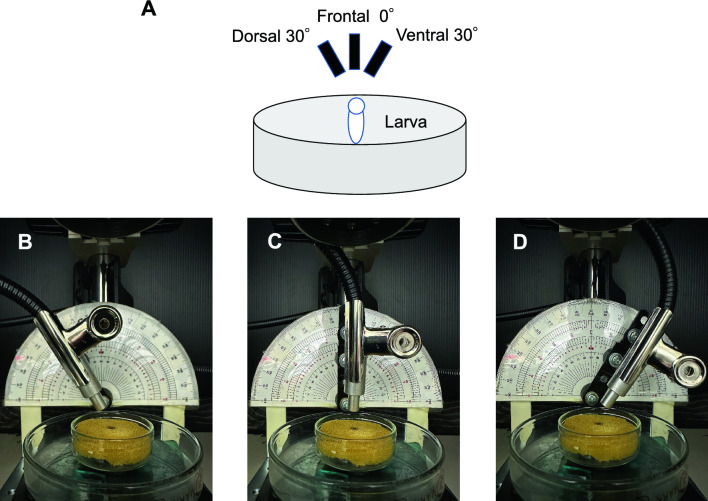
A schematic of light stimulation and figures of optical fiber cable position. (**A**) The position of larvae and light stimulation in three different directions. The position of optical fiber in different directions. (**B**), dorsal 30° (D30°); (**C**), frontal (F0°); (**D**), ventral 30° (V30°).

The stimulus light was derived from a white LED (NS6W083BT, NICHIA Corporation, Tokushima, Japan) using a fiber optic cable with an outer diameter of 8 mm and a core and clad of 5 mm. The tip of the fiber optic cable was placed 15 mm from the eye to allow uniform irradiation of the entire eye. The stimulation system was designed to deliver light stimulation from the frontal 0° (F0°), ventral 30° (V30°), and dorsal 30° (D30°) directions to the eye while keeping the distance between the optical fiber and the eye at 15 mm (Fig. 8). Based on our experience with previous ERG recordings from zebrafish larvae, we determined that the combined angle of both the angle of displacement from the front during the fixation of the larva to the recording table and the angle of displacement of the optical fiber would result in a maximum variation of 30°. The light intensity in each of the three directions was measured with a photometer (LumaColor™ Photometer J17 and Irradiance head J1812, SONY Tektronix, Tokyo, Japan). Stimulus light intensity was measured 10 times per direction. The differences in the light intensity in the three directions were not significant (*P* = 0.53; one-way ANOVA).

### Experimental procedures

All experiments were performed in the afternoon under room temperature of 28–30 °C. The age of the larvae was based on the dpf, and they were tested at the age of 5–6 dpf. The larvae were anesthetized by submersion in a solution of 0.02% 3-aminobenzoic acid methyl ester (MESAB; Sigma, St. Louis, MO, USA) in E3 medium until the swimming motions stopped. Then, they were paralyzed by submersion in a 0.8 mg/ml Esmeron (Organon Teknika, Eppelheim, Germany) solution in E3 medium. They were positioned on a moistened paper towel placed over a reference electrode in a Petri dish. The larvae were positioned with the right side up, head toward the back and tail toward the front, and the right eye facing forward (F0°) to the optical fiber set at 0°. Then, they were light-adapted to a background illumination of 10 mW/m^2^ for at least five minutes before the recordings. ERGs were recorded under photopic conditions. The stimulus intensity was fixed to 640 W/m^2^, the background luminance was 10 mW/m^2^, and the stimulus duration was 1000 ms. Five to ten responses were averaged with an inter-stimulus interval of three seconds. ERGs were first recorded at F0°, then at D30° after three minutes, and at V30° after another three minutes. From anesthesia to completion of recording, the duration of the experiment was approximately 40 min.

The amplitude of the b-wave was measured from the trough of the a-wave to the peak of the positive b-wave. The amplitude of the d-wave was measured from the baseline just before the d-wave to the peak of the d-wave. The implicit times of the b- and d-waves were measured from the stimulus onset to the peak of the b- and d-waves.

### Statistical analyses

Individuals with amplitudes less than 100 µV at F0° were excluded to rule out the possibility of small amplitudes due to congenital abnormalities. The descriptive data are presented as numbers, percentages, and means ± standard deviations (SD). One-way ANOVA with a post-hoc test using Holm–Šídák's multiple comparison test was used to determine the significance of the differences of the b- and d-wave amplitudes and implicit time between D30°, F0°, V30°. To determine the interindividual variations, the CVs of b- and d-wave amplitudes and implicit times at 0° among the larvae were calculated. All *P*-values were two-sided, and a *P* < 0.05 was taken to be statistically significant. The CV was classified as 'low' (< 10%), 'moderate' (≥ 10%, < 30%), and 'high' (≥ 30%). All statistical analyses were performed using Prism 9 software (GraphPad, Inc., La Jolla, CA, USA).

### Ethics statement

This study did not require approval by the Institutional Review Board of Mie University. The research was conducted in full compliance and strict accordance with the Association for Research in Vision and Ophthalmology (ARVO) Resolution on the Use of Animals in Ophthalmic and Vision Research. All experiments were performed in accordance with the ARRIVE guidelines and regulations of this committee.

## Data Availability

Data are available from the corresponding author upon reasonable request.
